# Cancer survival in England and the influence of early diagnosis: what can we learn from recent EUROCARE results?

**DOI:** 10.1038/sj.bjc.6605399

**Published:** 2009-12-03

**Authors:** C S Thomson, D Forman

**Affiliations:** 1 Cancer Research UK, Statistical Information Team, 61 Lincoln's Inn Fields, London WC2A 3PX, UK; 2 Centre for Epidemiology and Biostatistics, University of Leeds, Beckett Street, Leeds, UK; 3 National Cancer Intelligence Network, Queens House, 56 Lincoln's Inn Fields, London, UK

**Keywords:** EUROCARE, cancer survival in England, National Awareness and Early Diagnosis Initiative (NAEDI)

## Abstract

**Background:**

This review of the EUROCARE-4 results attempts to separate out the early and late mortality effects contributing to the widely reported poorer 5-year survival rates for cancer patients in the United Kingdom compared with other European countries for 26 cancer sites.

**Methods:**

Patients diagnosed with cancer in 1996–1999 in 23 European countries were included in the analyses. Comparison of 1-year, 5-year and 5∣1-year (i.e. only including those patients who had survived to 1 year) survival estimates between data for England and the ‘European average’ was undertaken. This analysis was to highlight the relative contribution of early diagnosis, using 1-year survival as a proxy measure, on 5-year survival for the different sites of cancer. Three groups of cancer sites were identified according to whether the survival differences at 1, 5 and 5∣1 years were statistically significant.

**Results and conclusions:**

Breast cancer showed significantly poorer 1- and 5-year survival estimates in England, but the 5∣1-year survival figure was not significantly different. Thus, successful initiatives around awareness and early detection could eradicate the survival gap. In contrast, the 5∣1-year survival estimates remained significantly worse for lung, colorectal and prostate cancers, showing that although early detection could make some difference, late effects such as treatment and management of the patients were also influencing long-term outcome differences between England and Europe.

It has been widely reported that 5-year survival rates for cancer patients in the United Kingdom have, in general, lagged behind comparator European countries (and the ‘European average’) since the late 1970s (Berrino *et al*, 1995b, 1999, 2003, 2007). The NHS Cancer Plan (Department of Health, 2000) highlighted this survival ‘gap’ and ‘catching up with Europe’ has been a major driver in formulating national cancer policy.

High-resolution studies of a few specific sites of cancer undertaken within some of the registries included in EUROCARE-2, and in which additional data on stage and diagnostic techniques were available, suggested that later stage at presentation among UK patients was a major factor explaining the poorer survival. These data are, however, now quite old; they relate to cancers diagnosed in the 1980s and have not been updated (Gatta *et al*, 2000; Sant *et al*, 2003, 2007).

The most recently published EUROCARE data, based on new diagnoses between 1995 and 1999, indicate that the survival gap between England and the ‘European average’ had diminished but had not been completely removed (Berrino *et al*, 2007; Richards, 2007; Verdecchia *et al*, 2007). As a consequence, the 2007 Cancer Reform Strategy (CRS) maintained an objective of bringing about service improvements to reduce further the difference in survival between England and Europe (Department of Health, 2007).

The CRS set out several initiatives to improve survival including establishment of the National Awareness and Early Diagnosis Initiative (NAEDI). One objective of NAEDI is to review the evidence on links between early diagnosis and survival and evaluate the extent to which international differences in survival are due to differences in delays in patient presentation; in practitioner referrals; in the availability and quality of diagnostic techniques used; and in the management and care of patients once diagnosed.

Use of 1-year survival as a proxy for early/late diagnosis, and hence for stage data, has been advocated in the CRS and comparison of this outcome measure across Europe may help benchmark the current disparities. However, the appropriateness of this measure will vary by cancer site and early/late presentation is not the only possible reason for observed differences. For example, for cancers such as oesophageal and pancreatic with a generally poor prognosis, the introduction of chemotherapeutic regimens for advanced disease will have improved 1-year survival rates for patients irrespective of the proportion of early stage tumours in the populations (Rao and Cunningham, 2008; Starling and Cunningham, 2008; Mitry *et al*, 2008a, 2008b). Also factors, such as differences in underlying comorbidity, will impact on short-term survival but are rarely adjusted for in comparative survival estimates.

Use of 5-year survival data conditional on surviving 1 year (i.e. 1–5 year or 5∣1 survival) has been presented in the most recent EUROCARE-4 publications (Sant *et al*, 2009). Examination of this along side 1-year survival for different cancer sites shows which sites had variation between the countries at 1 year, which was not present at 5 years after those who died within the first year had been excluded from the analyses. To some extent, this enables separation of the effects of stage distribution, significant comorbidity and either peri-operative (or postoperative treatment-related) mortality, shown generally by the 1-year survival results, from the effects of treatment and patient management on survival, shown by differences in longer-term survival. This assessment is fundamental to the NAEDI objective of trying to identify cancer sites where delays in diagnosis have had the biggest impact on survival and which could be targeted to reduce such delays in the future.

This paper analyses the most recent EUROCARE data for the more common sites of cancer, comparing outcomes in England with the EUROCARE-4 ‘European average’ at 5, 1 and 5∣1 years after diagnosis, and also looks at recent trends in 1-year survival data within England. This provides a means of beginning to identify cancer sites for which the poor outcomes in England can be attributed to later stage at diagnosis.

## Materials and methods

The EUROCARE project has been running since 1990 (Berrino *et al*, 1995a). The latest published data cover patients diagnosed between 1996 and 1999 and are based on 2.8 million adults (aged 15–99) who were diagnosed with cancer in 23 European countries (Sant *et al*, 2009). Data for 26 of the more common sites or site groups of cancer from the EUROCARE-4 study have been presented. The coding classification used in EUROCARE-4 is the third revision of the International Classification of Diseases for Oncology (ICD-O3).

The data presented here from the EUROCARE-4 study are the figures for England and the ‘European average’, which have both been appropriately standardised for cancer survival analyses. This was to take into account any differences in age structures between the countries, and four different standards are chosen depending on the cancer site of choice. The International Cancer Survival Standard (ICSS; Corazziari *et al*, 2004) was applied to the EUROCARE-4 data, except for prostate cancer (De Angelis *et al*, 2009; pp 20). This differs from previous EUROCARE studies, which used internally weighted average age structure for Europe for that study. The ICSS gives standard weights, which should make future comparisons easier.

Data from recent analyses within England (Rachet *et al*, 2009) for 20 cancer sites or site groupings have also been included. The classification used for this study was the tenth revision of the ICD-10, which is not entirely comparable with those coded using ICD-O3. These data were not originally age standardised although equivalent age-standardised rates were made available to us (personal communication, B Rachet, 2009). The age-standardised data were not reported in the original paper because the authors had found that the age distributions of the cancer patients had changed little over the 11 years they examined. However, the weights used in the age standardisation for the recent study in England (Rachet *et al*, 2009) were different to those used in the EUROCARE-4 publications. Instead of the ICSS weighting system, Rachet *et al* (2009) used the ‘England Standard’, which is based on the age distributions of cancer patients diagnosed between 1986 and 1990 in England (Coleman *et al*, 1999; pp 50). This was to maintain comparability with previous survival analyses within England and used a different set of weights for each of the 20 cancer sites.

The ‘European average’ survival rates reported are not strictly true averages across the whole of Europe. This is because only 13 of the 23 countries represented in EUROCARE-4 have national cancer registration and, hence, 100% coverage. The range of coverage for the other countries was from 1.3% of the adult population for Germany to 58% for Belgium. The data included in EUROCARE-4 covered a population of just over 150 million, which represented about 35% of the population of the combined countries taking part in the study, and 30% of the total EU population (excluding Norway, Switzerland and Iceland, which are not in the EU).

The methodology used to derive the ‘European average’ was neither a simple country-specific weighted average based on coverage, nor one based on population, because either of these averages would have been heavily biased. Instead, regional-specific survival estimates were obtained by splitting Europe into five areas and pooling the data available for the regions. This makes the assumption that the survival for the population covered by registration was representative of the unknown survival of the whole region. Weighting was then applied to each of these five regional estimates using the mean population for those countries included in EUROCARE-4 for each region.

The method of creating the ‘European average’ led to the United Kingdom and Ireland region making up 14.6% of the ‘European average’. Thus, comparison of the survival estimates for England (or indeed any other country) with the survival estimate for the ‘European average’ is not strictly straightforward, due to the non-independence between them. However, it was assumed that the covariance (a measure of the non-independence) between the England value and the ‘European average’ would not be large enough to greatly affect interpretation of statistical significance, and as such, the difference between the survival rates for England and the ‘European average’ for each of the 5-year, 1-year and 5∣1-year estimates was obtained, along with an estimate of its standard error (s.e.). The standard error for the survival difference was calculated by taking the square root of the sum of the squared standard errors obtained for each of the survival estimates for England and the ‘European average’, ignoring the covariance between the two cohorts.

## Results

Table 1 shows results for England and the ‘European average’ for 5, 1 and 5∣1-year survival results for 26 of the more common sites or site groups of cancer included in the EUROCARE-4 study. Five-year survival was significantly worse in England for 14 of these cancers, including the four major sites, breast, lung, colorectal and prostate, together with cancers of the oesophagus, stomach, liver, pancreas, cervix, ovary, kidney, brain, thyroid and multiple myeloma. Five-year survival was significantly better in England for head and neck cancers (including the group of oral cancers) and malignant melanoma; and not significantly different for the remaining 10 sites.

All of the 14 cancers showing a statistically significant worse outcome in England at 5 years also showed a significantly worse outcome at 1 year. A further five cancers – bone and cartilage, soft tissue, uterus, bladder and non-Hodgkin lymphoma – also showed a significantly worse 1-year survival, but this was not maintained at 5 years. Of the 14 cancers with significantly worse 5-year outcomes, eight also showed a significantly worse 5∣1-year survival – lung, colorectal and prostate together with stomach, ovary, kidney and thyroid cancers and multiple myeloma. The other six cancers had 5∣1-year survival that was no longer significantly different from the ‘European average’. This group included breast cancer together with oesophagus, liver, pancreas, cervix and brain cancers.

Table 2 shows the 14 cancers with significantly worse 5-year outcomes ordered by the magnitude of the 5-year ‘survival gap’ between England and the ‘European average’ together with the corresponding survival gap at 1 year and 5∣1 years. Kidney cancer showed the largest survival gap, being 12.4% worse than the ‘European average’ at 5 years, while four other sites had gaps that were larger than 5% worse (stomach (8.4%), prostate (6.7%), ovary (6.3%) and thyroid (5.3%)).

Of the sites for which a statistically significant gap was maintained for 5∣1-year survival, all except stomach cancer had a reduced gap in comparison with that observed from the conventional 5-year survival analysis. For stomach cancer, survival in England was 10.5% worse than the ‘European average’ in the 5∣1-year analysis (compared with 8.4% for the conventional 5-year outcome). Of the other sites, only kidney (6.0%) and prostate cancer (5.1%) now had gaps of more than 5%.

**Figure 1:**
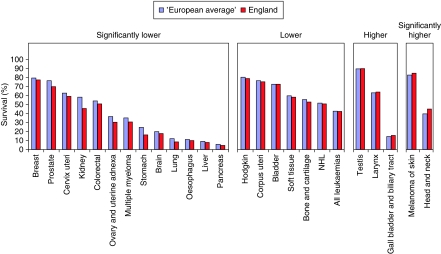
Five-year survival of patients diagnosed 1995–1999 from the EUROCARE-4 Study followed up to until the end of 2003, age-standardised % relative survival, ‘European average’ and England.

**Figure 2:**
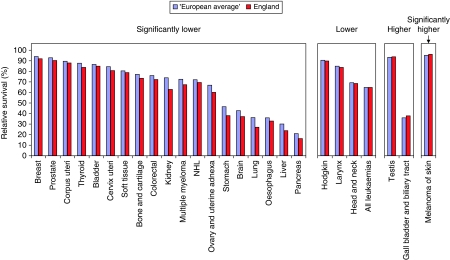
One-year survival of patients diagnosed 1995–1999 from the EUROCARE-4 Study followed up to until the end of 2003, age-standardised % relative survival, ‘European average’ and England.

**Figure 3:**
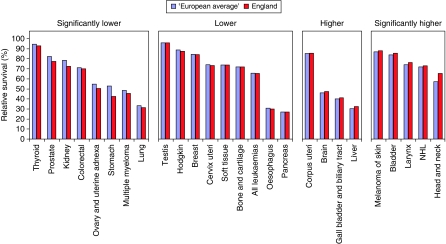
5∣1-year survival of patients diagnosed 1995–1999 from the EUROCARE-4 Study followed up to until the end of 2003, age-standardised % relative survival, ‘European average’ and England.

Figures 1, 2 and 3 provide graphical comparisons of the differences between England and the ‘European average’ at 5, 1 and 5∣1 years.

Table 3 provides a crude comparison between the EUROCARE-4 data, for both England and the ‘European average’ for patients diagnosed in 1995–1999, with the 1-year survival rates for England between 1996 and 2000 reported in 2009, together with data for 2004–2006 showing changes in 1-year survival rates in England between these two time periods. Due to the different methodologies used, direct comparison between EUROCARE-4 and the more recent England data is not strictly possible but, as expected, for most of the cancers considered, the England results from EUROCARE-4 are fairly similar to the more recent analysis of England data for 1996–2000 (the major exception being ovarian cancer for which the EUROCARE-4 analysis provides a 1-year survival of 59.9% while the more recent analysis for 1996–2000 estimates 69.1%).

Of the 20 individual cancer sites or site groups considered in Table 3 for England, 17 have shown an improvement in 1-year survival between 1996–2000 and 2004–2006 (the exceptions being cervical cancer (0.1% decrease), Hodgkin disease (1.4% decrease) and leukaemia (2.3% decrease)). These improvements were generally around 1–2% between the two time periods and the major cancers, breast, lung, colon, rectum and prostate all showed survival increases of this magnitude. For testicular cancer, 1-year survival in 1996–2000 was already extremely high (97.8%) and the improvement, by 0.1%, was marginal. The improvements in oesophageal (5.0%), stomach (5.3%) and brain cancer (4.4%) were the most substantial.

## Discussion

Until recently, the only evidence enabling an understanding of the reasons for the relatively poor 5-year survival outcomes in the United Kingdom compared with elsewhere in Europe was the EUROCARE high-resolution studies (Gatta *et al*, 2000; Sant *et al*, 2003, 2007). While these indicated later stage at diagnosis as being an important explanation for part of the difference, the studies only covered a few sites of cancer and were based on relatively small numbers of patients diagnosed during the early 1980s. The samples of UK patients included in these were not necessarily representative of either their regions of residence, or the country as a whole. Relevance to the current situation is, therefore, questionable.

The present review of the EUROCARE-4 results cannot provide an exhaustive evaluation of the relative contribution of stage at diagnosis to 5-year survival on a site-by-site basis but the analysis of 5, 1 and 5∣1-year survival helps provide some overall perspective.

Of the cancer sites considered, one important group is those cancers for which survival in England was significantly worse than the ‘European average’ at 5 years, but separating this into outcomes after 1 year and between 1 and 5 years (i.e. having survived for 1 year) indicated a statistically significant detriment associated only with the former, but not the latter. This implies that, for these cancers, it is low survival in the first year after diagnosis, which gives rise exclusively to the relatively poor overall outcome. The major site falling into this category was breast cancer, but others in this group were cancers of the oesophagus, liver, pancreas, cervix and brain. It is noteworthy that this group includes both breast and cervical cancers for which the benefit of population-based screening has long been established. Both of these cancers are associated with a generally good prognosis and the poor comparative survival in England is probably best explained by relatively small subgroups of women diagnosed with late stage disease, and not being detected by screening.

In contrast, the results for oesophageal, pancreatic and liver cancers, all with an extremely poor prognosis, may be explained in part by differences in access to new therapeutic regimens for advanced disease, which may have started use in England later than elsewhere in Europe (Mitry *et al*, 2008a, 2008b; Rao and Cunningham, 2008; Starling and Cunningham, 2008; Rimassa and Santoro, 2009). As a group, however, these cancers are often diagnosed at an advanced, incurable stage. If registration and follow-up of cases registered initially through death certificates were more effective in England than elsewhere, this would result in other countries having a higher proportion of cases that are more likely to be registered only through death certification and/or as cancers with an unknown primary site. These cases have very poor outcomes but need to be excluded from survival analyses. This would result in the poorer 1-year survival observed in England, but would not influence the 5∣1-year survival. Finally brain cancer, associated with moderate prognosis, is a type of cancer with a potential for early diagnosis if prompt attention is paid to symptoms and appropriate diagnostic facilities are available. With the exception of cervical cancer (marginal decrease) and liver cancer (not included in the analysis by Rachet *et al*, (2009)), all of the cancers in this group have shown recent improvements in 1-year survival in England (Table 3).

A second group of cancers comprises those that showed significantly lower survival at 1 year in England compared with the ‘European average’, which was no longer evident at either 5 or 5∣1 years. The five cancers in this group were bone and cartilage, soft tissue, uterus, bladder and non-Hodgkin lymphoma and, as with the preceding group, differences in early diagnosis and/or initial management of advanced disease would explain the disadvantage observed in England compared with the ‘European average’, although this is not translated into a differential at 5 years. All of these cancers are associated with reasonable survival rates overall and thus might benefit most from efforts towards earlier diagnosis. With bladder cancer, there is also the additional problem that changes in coding schemes defining the classification of invasive tumours and introduced at different times in different countries, may introduce artefactual survival differences.

The final group of eight cancers are those that had significantly lower survival rates in England at 5 years, which were also evident at both 1 year and 5∣1 years. This group included the three common cancers of the lung, colorectum and prostate, as well as cancers of the kidney, thyroid, ovary and stomach and multiple myeloma. The results for these cancers suggest that improving the stage at diagnosis of these cancers would not have eradicated all of the variation observed in 5-year survival between England and Europe. There may also have been differences in the treatment given or the management of services that affected the longer-term outcome. However, the survival gap for all of these sites, except stomach, was smaller between 1–5 years than it was between diagnosis and 5 years (Table 2) indicating a role for earlier diagnosis. In addition, all of these cancers (except thyroid that was not analysed by Rachet *et al*, 2009) have shown recent improvements in 1-year survival in the England data (Table 3). Prostate cancer survival results are particularly difficult to interpret given the unknown impact of differing intensity of PSA testing in different countries leading to overdiagnosis of non-fatal disease. Coding changes for the classification of invasive behaviour for ovarian cancer make comparisons over time problematic and are most apparent when using the different coding classifications ICD-O3 and ICD-10, as in Table 3.

This analysis dissects the EUROCARE-4 results to discriminate, among those cancers for which 5-year survival in England seemed to be lagging behind Europe, those for which the explanation seems to be largely due to effects within the year after diagnosis (group one, not showing detrimental 5∣1--year outcomes) and those for which the explanation is related to short- and longer-term effects (group three, showing detrimental 1 and 5∣1--year outcomes). There is also a group of cancers for which 5-year survival in England was not statistically different from the ‘European average’, but for which there was a detrimental effect at 1 year (group two). It should also be noted that, for 10 of the 26 cancer sites listed in Table 3, there was no statistical difference between the England outcomes and the ‘European average’ at 5 years, and for only two cancers (head and neck and malignant melanoma), England had significantly better survival than Europe.

Efforts and interventions directed towards increased cancer awareness and earlier detection, if successful, are likely to benefit all forms of cancer. Relative improvements to 1-year survival in England are likely to benefit those cancers in groups one and two to such an extent that the survival gap with Europe could almost completely be overcome. NAEDI could make this improvement to most of the cancers in these two groups although, for the poor prognosis sites (oesophageal, stomach and liver), life-extending therapies for advanced disease must also have a role. NAEDI could also have an important function in the cancers in group three, although the current survival gap between England and Europe for these cancers is not fully explained by short-term survival differences and other factors will thus also have a role.

This study has several limitations. It is acknowledged that the non-independence between the survival estimates for England and the ‘European average’ means that the method of testing differences between the survival estimates, using only the pooled variance to highlight significant differences, is not perfect. It should, however, be sufficient to help identify which sites require further efforts to separate out the early and late mortality effects and where future international benchmarking projects should be considered.

De Angelis *et al* (2009) describe the data quality and completeness across the EUROCARE-4 data sets, presenting indicators of quality known to affect survival analyses. One of these measures is the percentage of registrations made solely on the basis of death certificates (DCOs), which are excluded from the survival calculations thus biasing the results. High DCO rates are associated with over-inflation of the survival estimates (Berrino *et al*, 1995a), and the higher DCO rates in England compared with Europe (6.1% compared with 2.7%) suggest this is a factor which needs consideration and a possible correction could be applied to survival estimates (Silcocks and Thomson, 2009).

The concept of the ‘European average’ is itself problematic mainly because the EUROCARE-4 Study only covers a minority (about 30%) of the EU population. From the variation within the countries with partial coverage, it is likely that the national estimates for some of these countries would change if 100% population coverage were available, thus, in turn, affecting the ‘European average’. Another problem with the ‘European average’ is that it assumes that all the data sets used in its estimation are of equal quality in terms of accuracy and completeness of recording both incident cases and subsequent deaths. Further discussion of this is provided in both Møller *et al* (2009) and Abdel-Rahman *et al* (2009).

An alternative to using the ‘European average’ for comparison with England would be to identify specific populations with which to compare the results from England; these are likely to be with countries where comparable quality of data to those in England was assured. This is the basis of the future proposed international benchmarking studies.

These results also only give a crude assessment of the possible effects of stage, early diagnosis and treatment on outcomes. A better way to examine these effects is to include this information, when it has been collected robustly, directly in the survival analyses. The high-resolution studies, undertaken as part of the earlier EUROCARE studies, attempted this but, partly because of resource constraints involved in data collection, are not available for contemporary comparisons.

Another weakness currently faced is that though it is known how survival has improved in England during the past decade, how this compares with improvements made across Europe is not known. It is possible that the gaps are closing but have not been fully eradicated, and without further studies this cannot be determined for certain (Berrino *et al*, 2007; Richards, 2007; Verdecchia *et al*, 2007). This study has focussed on comparing survival within England with the ‘European average’. It could be argued that as a rich Western European country, England should be striving to be the equivalent of the ‘European best’ not just as good as the ‘European average’. This was the adopted approach for estimating the numbers of avoidable deaths in the United Kingdom relative to the ‘European best’ (Abdel-Rahman *et al*, 2009).

There is, therefore, a need for new international benchmarking studies to assess this. These studies will need to compare registry data within England with other countries with good quality registry data. The data collected will need to contain good staging information, which has been robustly collected to agreed standards, along with information about the diagnostic techniques used, the presence of any significant comorbidity and details of the treatment that was given. Only then may studies provide the real understanding as to how much stage at diagnosis and earlier detection improve cancer survival.

## Conflict of interest

The authors declare no conflict of interest.

## Figures and Tables

**Table 1:** Patients diagnosed 1995–1999 from the EUROCARE-4 Study followed up to until the end of 2003, age-standardised % relative survival (5-year, 1-year and 5∣1-year)

	**% 5-year survival**			**% 1-year survival**			**% 5∣1-year survival**		
**Site^a^**	**‘European average’**	**England EC4 1995–1999**	**England–‘Europe Avg’^b^**	**‘European average’**	**England**	**England–‘Europe Avg’^b^**	**‘European average’**	**England**	**England–‘Europe Avg’^b^**
Head and neck	39.5	44.8	Sig higher	69.1	68.5	Lower	57.2	65.3	Sig higher
Oesophagus	11.1	9.9	Sig lower	35.8	32.9	Sig lower	30.9	30.1	Lower
Stomach	24.5	16.1	Sig lower	46.3	38.0	Sig lower	52.9	42.4	Sig lower
Colorectal	54.0	50.5	Sig lower	75.8	72.2	Sig lower	71.2	70.0	Sig lower
Liver	9.1	7.7	Sig lower	30.0	23.7	Sig lower	30.2	32.5	Higher
Gallbladder and biliary tract	14.4	15.6	Higher	35.9	37.7	Higher	40.1	41.2	Higher
Pancreas	5.7	4.4	Sig lower	20.9	16.2	Sig lower	27.1	27.0	Lower
Larynx	62.8	63.9	Higher	84.7	83.7	Lower	74.1	76.4	Sig higher
Lung	12.0	8.4	Sig lower	36.0	26.9	Sig lower	33.3	31.3	Sig lower
Bone and cartilage	55.5	52.6	Lower	77.0	73.2	Sig lower	72.0	71.9	Lower
Soft tissue	59.5	58.0	Lower	80.4	78.6	Sig lower	73.9	73.8	Lower
Melanoma of skin	82.6	84.6	Sig higher	95.0	96.0	Sig higher	87.0	88.1	Sig higher
Breast^c^	79.4	77.3	Sig lower	93.8	91.8	Sig lower	84.6	84.2	Lower
Cervix uteri	62.6	59.1	Sig lower	84.4	80.6	Sig lower	74.1	73.3	Lower
Corpus uteri	76.2	75.2	Lower	89.5	87.9	Sig lower	85.2	85.6	Higher
Ovary and uterine adnexa	36.5	30.2	Sig lower	66.7	59.9	Sig lower	54.7	50.5	Sig lower
Prostate	76.4	69.7	Sig lower	92.7	90.2	Sig lower	82.4	77.3	Sig lower
Testis	89.5	89.7	Higher	93.3	93.7	Higher	96.0	95.8	Lower
Bladder	72.4	72.4	Lower	86.4	84.7	Sig lower	83.8	85.5	Sig higher
Kidney	58.0	45.6	Sig lower	73.8	62.7	Sig lower	78.6	72.6	Sig lower
Brain	19.7	17.6	Sig lower	42.7	37.1	Sig lower	46.0	47.4	Higher
Thyroid	82.9	77.6	Sig lower	87.6	83.7	Sig lower	94.6	92.8	Sig lower
Hodgkin	80.1	78.6	Lower	90.4	89.7	Lower	88.7	87.6	Lower
NHL	51.5	50.7	Lower	71.8	69.4	Sig lower	71.8	73.1	Sig higher
Multiple myeloma	35.1	30.6	Sig lower	72.3	67.1	Sig lower	48.6	45.6	Sig lower
All leukaemias	42.4	42.3	Lower	64.7	64.6	Lower	65.6	65.4	Lower

a Site groups classified according to ICD-O3 coding.

b ‘Sig lower’=England had significantly poorer survival than ‘European average’; ‘Sig higher’=England had significantly better survival than ‘European average’. Non-significant differences were labelled as either ‘Lower’ or ‘Higher’ based on the point estimates for England compared with ‘European average’.

c Based on persons.

**Table 2:** Patients diagnosed 1995–1999 from the EUROCARE-4 Study followed up to until the end of 2003, % difference in relative survival (5-year, 1-year and 5∣1-year) for the 14 sites with significantly lower 5-year survival

	**% difference in 5-year survival**	**% difference in 1-year survival**		**% difference in 5∣1-year survival**	
**Site^a^**	**England–‘Europe Avg’**	**England–‘Europe Avg’**	**Difference^b^**	**England–‘Europe Avg’**	**Difference^b^**
Kidney	−12.4	−11.1	Sig Lower	−6.0	Sig Lower
Stomach	−8.4	−8.3	Sig Lower	−10.5	Sig Lower
Prostate	−6.7	−2.5	Sig Lower	−5.1	Sig Lower
Ovary and uterine adnexa	−6.3	−6.8	Sig Lower	−4.2	Sig Lower
Thyroid	−5.3	−3.9	Sig Lower	−1.8	Sig Lower
Multiple myeloma	−4.5	−5.2	Sig Lower	−3.0	Sig Lower
Lung	−3.6	−9.1	Sig Lower	−2.0	Sig Lower
Colorectal	−3.5	−3.6	Sig Lower	−1.2	Sig Lower
Cervix	−3.5	−3.8	Sig Lower	−0.8	Lower
Breast^c^	−2.1	−2.0	Sig Lower	−0.4	Lower
Brain	−2.1	−5.6	Sig Lower	1.4	Higher
Liver	−1.4	−6.3	Sig Lower	2.3	Higher
Pancreas	−1.3	−4.7	Sig Lower	−0.1	Lower
Oesophagus	−1.2	−2.9	Sig Lower	−0.8	Lower

a Site groups classified according to ICD-O3 coding.

b ‘Sig lower’=England had significantly poorer survival than ‘European average’; ‘Sig higher’=England had significantly better survival than ‘European average’. Non-significant differences were labelled as either ‘Lower’ or ‘Higher’ based on the point estimates for England compared with ‘European average’.

c Based on persons.

**Table 3:** Age-standardised relative survival (1-year) for patients diagnosed 1995–1999 for England and the ‘European average’ followed up to the end of 2003 (EUROCARE-4 Study); and patients diagnosed 1996–2000 and 2004–2006 in England (Rachet p. comm) followed up to the end of 2007

**Site^a^**	**‘European average’ 1995–1999 EUROCARE-4 (ICSS)**	**England 1995–1999 EUROCARE-4 (ICSS)**	**England 1996–2000 (Rachet) (England Standard)**	**England 2004–2006 (Rachet) (England Standard)**
Breast	93.8^b^	91.8^b^	93.9^c^	95.5^c^
Lung	36.0	26.9	27.0^d^	28.9^d^
Colon	74.2	69.9	69.5^d^	71.8^d^
Rectum	78.7	76.3	76.1^d^	78.1^d^
Prostate	92.7	90.2	91.5	93.3
Oesophagus	35.8	32.9	32.8^d^	37.8^d^
Stomach	46.3	38.0	35.8^d^	41.1^d^
Pancreas	20.9	16.2	14.7^d^	16.8^d^
Larynx	84.7	83.7	83.7^e^	85.8^e^
Melanoma	95.0	96.0	95.5^d^	96.6^d^
Cervix	84.4	80.6	83.0	82.9
Corpus uteri	89.5	87.9	88.3	90.1
Ovary and uterine adnexa	66.7	59.9	69.1	71.3
Testis	93.3	93.7	97.8	97.9
Kidney	73.8	62.7	67.2^d^	69.7^d^
Brain	42.7	37.1	33.7^d^	38.1^d^
NHL	71.8	69.4	71.6^d^	75.2^d^
Hodgkin	90.4	89.7	91.3^d^	89.9^d^
Multiple myeloma	72.3	67.1	65.4^d^	66.3^d^
All leukaemias	64.7	64.6	63.4^d^	61.1^d^

a Site groups classified according to ICD-O3 coding for EUROCARE-4 but using ICD-10 coding for the England analyses by Rachet *et al*, 2009.

b Based on persons.

c Based on females only.

d Estimates taken as simple averages of the male and female estimates.

e Based on males only.

ICSS=The International Cancer Survival Standard; ICD-O3=International Classification of Diseases for Oncology.
